# Species-Related Differences in the Proteome of Rat and Human Pancreatic Beta Cells

**DOI:** 10.1155/2015/549818

**Published:** 2015-05-10

**Authors:** G. A. Martens

**Affiliations:** ^1^B-Probe, Diabetes Research Center, Brussels Free University (VUB), Belgium; ^2^Department of Clinical Chemistry & Radioimmunology, University Hospital Brussels, Laarbeeklaan 103, 1090 Brussels, Belgium

## Abstract

The core proteomes of human and rat pancreatic beta cells were compared by label-free LC-MS/MS: this resulted in quantification of relative molar abundances of 707 proteins belonging to functional pathways of intermediary metabolism, protein synthesis, and cytoskeleton. Relative molar abundances were conserved both within and between pathways enabling the selection of a housekeeping network for geometric normalization and the analysis of potentially relevant differential expressions. Human beta cells differed from rat beta cells in their lower level of enzymes involved in glucose sensing (MDH1, PC, and ACLY) and upregulation of lysosomal enzymes. Human cells also expressed more heat shock proteins and radical scavenging systems: apart from SOD2, they expressed high levels of H_2_O_2_-scavenger peroxiredoxin 3 (PRDX3), confirmed by microarray, Western blotting, and microscopy. Besides conferring lower susceptibility to oxidative stress to human cells PRDX3 might also play a role in physiological redox regulation as, in rat, its expression was restricted to a beta cell subset with higher metabolic glucose responsiveness. In conclusion, although their core proteomic architecture is conserved, human and rat beta cells differ in their molar expression of key enzymes involved in glucose sensing and redox control.

## 1. Introduction

Most of our current understanding of the physiology of pancreatic beta cells comes from studies in rodents. These led to the elucidation of conserved biochemical signaling systems in control of nutrient-regulated insulin production [1] and conserved clusters of beta cell-selectively expressed genes, with role in endocrine function [2]. Yet rodents and humans differ dramatically in circadian rhythm, feeding behavior, and life span, imposing different physiological demands on the beta cells. Previous studies indicated that human beta cells display a higher glucose responsiveness than rodent beta cells [1] and a higher resistance to reactive oxygen species (ROS) and, unlike rodent beta cells [3], they are considered long-lived and postmitotic in adults [4, 5]. These functional differences are reflected by adaptations in their gene/protein expression patterns, for example, by a higher expression of heat shock protein 70 (HSP70) and some ROS scavenging enzymes (heme oxygenase 1, catalase, and superoxide dismutase 2) [6–8]. Evidently, quantitative comparison of protein expression levels between species is tricky and prone to biases introduced by differences in the affinity of antibodies to protein homologs, lack of attention for the normalization strategy, and inevitable variations in the cellular composition of isolated cell populations. Here, I attempted to overcome some of these challenges, by using label-free liquid chromatography-tandem mass spectrometry (LC-MS/MS) for antibody-independent quantification of molar protein abundances, geometrically normalized [9] towards a mininetwork of 6 conserved reference proteins, on unfractionated protein extracts from rat and human beta cell preparations with known endocrine purity. I quantified the proteomes of pancreatic islets obtained from 4 human adults with the same technique previously used to quantify the proteomes of FACS-purified rat alpha and beta cells [10, 11]. Within the constraints imposed by the depth of proteome coverage, I could thus study the relative molar abundances of core functional pathways within human beta cell preparations and directly compare these to rat beta and alpha cells. I found that the expression levels of core functional pathways (intermediary metabolism, protein synthesis, signaling, and cytoskeleton) were well conserved but also discovered interesting differences, particularly in enzymes of nutrient metabolism and antioxidant defenses.

## 2. Materials and Methods

### 2.1. Beta Cell Isolation

Rats were housed according to the Belgian animal welfare regulations. Animal killing was kept to the strict minimum, after proper CO_2_-anesthesia. Use of animal cells and tissues was approved by the Commissie Proefdiergebruik (CPG) of the Vrije Universiteit Brussel (VUB), for a project entitled “In Vitro and In Vivo Markers for Beta Cell Death and Function” (CPG approval ID 07-274-3). Rat beta and islet nonbeta cells were isolated from healthy 10-week-old, fed ad libitum on a cereal-based standard chow (Scientific Animal Food & Engineering, SAFE—A04 maintenance diet) and housed at normal day/night cycles. Isolated endocrine preparations consisted of ≥95% endocrine cells and <2% exocrine cells. Beta cell preparations consisted of 90% insulin+, 3% glucagon+, 1% somatostatin+, and 2% pancreatic polypeptide+ cells; alpha cells contained 2% insulin+, 94% glucagon+, 1% somatostatin+, and 2% pancreatic polypeptide+ cells, as previously described [10, 12]. Human beta cells were used for research after approval by the Ethical Committee of the Universitair Ziekenhuis Brussel (BUN 143201213515 and BUN 14320109289). They were obtained by dissociation of islet-enriched human pancreas fractions, followed by FACS-sorting according to cell size, granularity, and zinc content, to an average insulin-positive purity of 60 ± 6% (range: 53–68%) with 13 ± 8% glucagon-positive cells and 21 ± 7% nongranulated cells. Beta cells were isolated from nondiabetic donors, age (XXX), and BMI (XXX).

### 2.2. Protein Extraction and Trypsinization

Cells were washed 3 times with PBS (4°C) and soluble protein was extracted in 50 *μ*L 0.5% (w/v) RapiGest detergent in 50 mM ammonium bicarbonate (Waters Corporation, Milford, MA) in the presence of Complete Protease Inhibitor Cocktail (F. Hoffmann–La Roche Ltd, Basel, Switzerland) and bovine DNAse II (Boehringer Ingelheim GmbH, Ingelheim am Rhein, Germany, 2 *μ*g/mL) solution, followed by centrifugation (10,000 ×g, 10 min) to remove cellular debris. 25 *μ*L protein extract was reduced with 2.5 *μ*L 100 mM dithiothreitol (DTT) at room temperature for 60 min, followed by 3 subsequent washes with 400 *μ*L 50 mM ammonium bicarbonate and 4 *μ*L 100 mM DTT using a 5 KDa cut-off membrane filter. This step removes protease inhibitors and most of reduced insulin molecules. Proteins were denatured by heating at 80°C for 15 min, followed by 30 min at 60°C after addition of 2.5 *μ*L 100 mM DTT and another 30 min at ambient temperature in the dark after addition of 2.5 *μ*L 200 mM iodoacetamide. Trypsinization was carried out overnight at 37°C (1 : 25 w/w trypsin ratio) in final volume of 100 *μ*L. Finally, RapiGest detergent was removed by acidifying digest to pH = 2 with trifluoroacetic acid and incubation for 15 min at 37°C.

### 2.3. LC-MS Configuration

Nanoscale LC separation of the tryptic peptides was performed with a NanoAcquity system (Waters). Samples were loaded on to a Symmetry C18 5 *μ*m, 2 cm × 180 *μ*m trap column (Waters) at a flow rate of 5 *μ*L/min prior to separation on a Bridged Ethyl Hybrid C18 1.7 *μ*m, 25 cm × 75 *μ*m analytical reversed-phase column (Waters) by application of a 90 min gradient from 1% ACN and 0.1% formic acid to 40% ACN and 0.1% formic acid at a column flow rate of 0.25 *μ*L/min. The column temperature was maintained at 35°C. Analysis of the eluted tryptic peptides was performed using a Synapt G2 Q-TOF (quadrupole time-of-flight) mass spectrometer (Waters) equipped with a nanolockspray source (Waters) fitted with a pico-tip emitter (New Objective) operated at a capillary voltage of approximately 3 kV. For all measurements, the mass spectrometer was operated in v-mode with a typical resolution of at least 20,000 full width at half maximum. All analyses were performed in positive mode ESI. The time-of-flight analyzer of the mass spectrometer was externally calibrated with a NaI mixture from *m*/*z* 50 to 1990. The collision gas used was argon, maintained at a constant pressure of 2.0 × 10^−3^ mbar (1 bar = 100 kPa) in the collision cell. The lock mass, [Glu1]fibrinopeptide B, was delivered from the auxiliary pump of the NanoAcquity system with a concentration of 100 fmol/*μ*L at 0.5 *μ*L/min to the reference sprayer of the nanolockspray source. The data were postacquisition lock-mass corrected using the monoisotopic mass of the doubly charged precursor of [Glu1]fibrinopeptide B, delivered through the reference sprayer, which was sampled every 120 s. Accurate mass precursor and fragment ion LC-MS data were collected in data independent, alternate-scanning (LC-MSE) mode of acquisition [13, 14].

### 2.4. LC-MS Data Processing and Protein Identification

Continuum LC-MS data were processed and searched using ProteinLynx GlobalSERVER v2.5 (Waters Corporation). Protein identifications were obtained by searching databases of Rattus norvegicus databases (v15.12, 7,449 entries) and Homo Sapiens release (2011_11, 20335 entries). Sequence information of Alcohol dehydrogenase Saccharomyces cerevisiae was added to the databases to afford the ability to normalize the data sets and to estimate amounts and concentration and that of known contaminant proteins (e.g., serum albumin* Bos taurus* and trypsin* Sus scrofa*). A decoy was generated on the fly with every database search experiment conducted to estimate the protein false positive rate of identification. Data independent scanning protein identifications were accepted when more than three fragment ions per peptide, seven fragment ions per protein, and more than 2 peptides per protein were identified, in at least one technical replicate per sample. Protein quantitation was only reported when the protein was detected in at least 2 out of 3 technical replicates of at least one biological replicate. Typical search criteria used for protein identification included automatic peptide and fragment ion tolerance settings (approximately 10 and 25 ppm, resp.), 1 allowed missed cleavage, fixed carbamidomethyl-cysteine modification, and variable methionine oxidation. Raw data were expressed as “relative molar amount units” calculated by dividing the determined molar amount for a given protein by the summed determined amount for all identified proteins as this accounts for both technical and biological variations [10, 14, 15].

### 2.5. Selection of a Reference Protein Network

To allow quantitative comparison of proteomes between cell types, a set of reference or “housekeeping” proteins was selected for geometric normalization [9] (see Figure S1 in Supplementary Material available online at http://dx.doi.org/10.1155/2015/549818). A reference protein was defined by its detection in all biological replicates of all studied cell types, at a quantitatively stable level. Within each cell type, I first calculated matrices displaying the relative expression ratio of each candidate housekeeping protein, references to all the others, and then calculated the coefficient of variation (CV%) on all these ratios across different samples. Any given pair of proteins was considered stably regulated if the CV% on their expression ratio was ≤35% (arbitrary cutoff). I then arbitrarily assigned a score of 1 or 0 to protein pairs with CV%, respectively, ≤ or >35%. Summation of these scores, expressed as percent of maximal score achievable (102 for rat and 50 for human), was used to calculate a “matrix correlation score,” denoting the degree of overall “connectivity” or expression stability towards the other candidate housekeeping (Figures S1B-C). In the human data set, 51 proteins could thus be quantified in all samples: these ubiquitously LC-MS/MS-detectable proteins are evidently enriched in the abundant, “housekeeping” functional pathways of the cell: intermediary metabolism, protein synthesis, cytoskeleton, and signaling scaffolds (Figure S1.D). As expected, proteins belonging to the same pathway (e.g., glycolysis) show overall good correlation (Figure S1.D, red CV% <35%), reflecting their regulated coexpression, but they also show quantitatively stable expression with other functional pathways: for example, the molar expression level of the Krebs cycle enzyme malate dehydrogenase 2 (MDH2) is relatively stable not only towards enzymes of the upstream glycolysis (ALDOA, TPI1, GAPDH, and PGK1), but also to the amount of downstream mitochondrial F0F1 ATPase (ATP5A1), and even to unrelated functional pathways such as proteins synthesis/turnover (ubiquitin C, UBC, and heat shock protein A8, HSPA8), cytoskeleton (profilin 1, PFN1), and cell signaling (calmodulin 1, CALM1). Iteration of this analysis on all the rat samples yielded 103 proteins that could be quantified in all samples (Figure S1). Merging rat and human data resulted in 26 candidate references common to both species (shown in Figure S1.C). From these, I handpicked a final set of 6 reference proteins, integrating the following considerations: (i) showing among them a high degree of coregulation or “connectivity”; (ii) being selected from different functional pathways such as metabolism: MDH2, protein synthesis/turnover: UBC, HSPA8, cyclophilin-A/peptidyl prolylisomerase (PPIA), cytoskeleton (PFN1), and cell signaling (CALM1), and (iii) historical use as reference mRNA and/or protein (UBC, PPIA). Processed raw LC-MS/MS data (relative molar amount units) were then geometrically normalized for this network of 6 references as described and referred to GEO6 [9, 10]. These data reveal remarkably similar proteome architecture across species in terms of their reference proteins: for example, UBC is typically 1.9 ± 0.4- and 2.6 ± 0.4 times more abundant than PPIA and CALM1, respectively, both in human and rat beta cell preps (Figure S2.A). To illustrate that geometric normalization with several reference proteins achieves accurate quantification and allows direct comparison or protein abundances between rat and human beta cell preps, I verified the stoichiometry of a classical multiprotein complex: the mitochondrial F0F1 ATPase (Figure S2.B): the geometrically normalized molar abundances of subunits ATP5D, ATP5O, ATP5F1, and ATP5H all occur at approximately 1 : 3 to the abundances of subunits ATP5A1 and ATP5B, in line with the predicted stoichiometry [16].

### 2.6. Statistical Discrimination between Samples Based on Estimated Total Error

Total imprecision, including technical and biological variations, is ±19% [10]. To determine the statistically acceptable discrimination limit between two samples, a normal distribution of the proteome data was assumed. Full width at half maximum (FWHM) of these distributions was chosen as statistical discrimination limit to assess protein expression—a value corresponding to 2.35 times the median total error (19% times 2.35 = 45%).

### 2.7. Specific Reagents

Immunofluorescence and Western blot analysis of PRDX3 was done using a rabbit polyclonal raised against full-length human PRDX3, not cross-reactive to other PRDX isoforms (Abcam, UK, Ab15573). Immunofluorescence was done after deparaffinization of 5 *μ*M rat and human pancreas sections (*n* = 3) heat induced antigen retrieval without buffer, after overnight incubation with anti-PRDX3 at 1 : 1000, followed by 1 h using Cy3 anti-rabbit (Jackson Immunoresearch Laboratories, UK, 711-166-152) at 1 : 500. For Western blotting, total protein was extracted from archived (−80°C) human and rat tissue using RIPA with Roche Protease Inhibitor Cocktail and 10 *μ*g total protein extract was loaded. Membranes were first stained using 1 : 10000 anti-PRDX3 and anti-rabbit IR Dye 800 CW 1 : 10000 (Li-cor Biosciences, USA, 926-32211). Membranes were then stripped and stained a second time to verify proper loading using 1 : 1000 antibody against cyclophilin-A/PPIA (Abcam, UK, Ab41684) as reference. MDH1 was visualized using Abcam ab76616 mouse monoclonal raised against a recombinant human MDH1 fragment.

## 3. Results

### 3.1. Depth of Proteome Coverage and General Data Description

**Figure 1:**
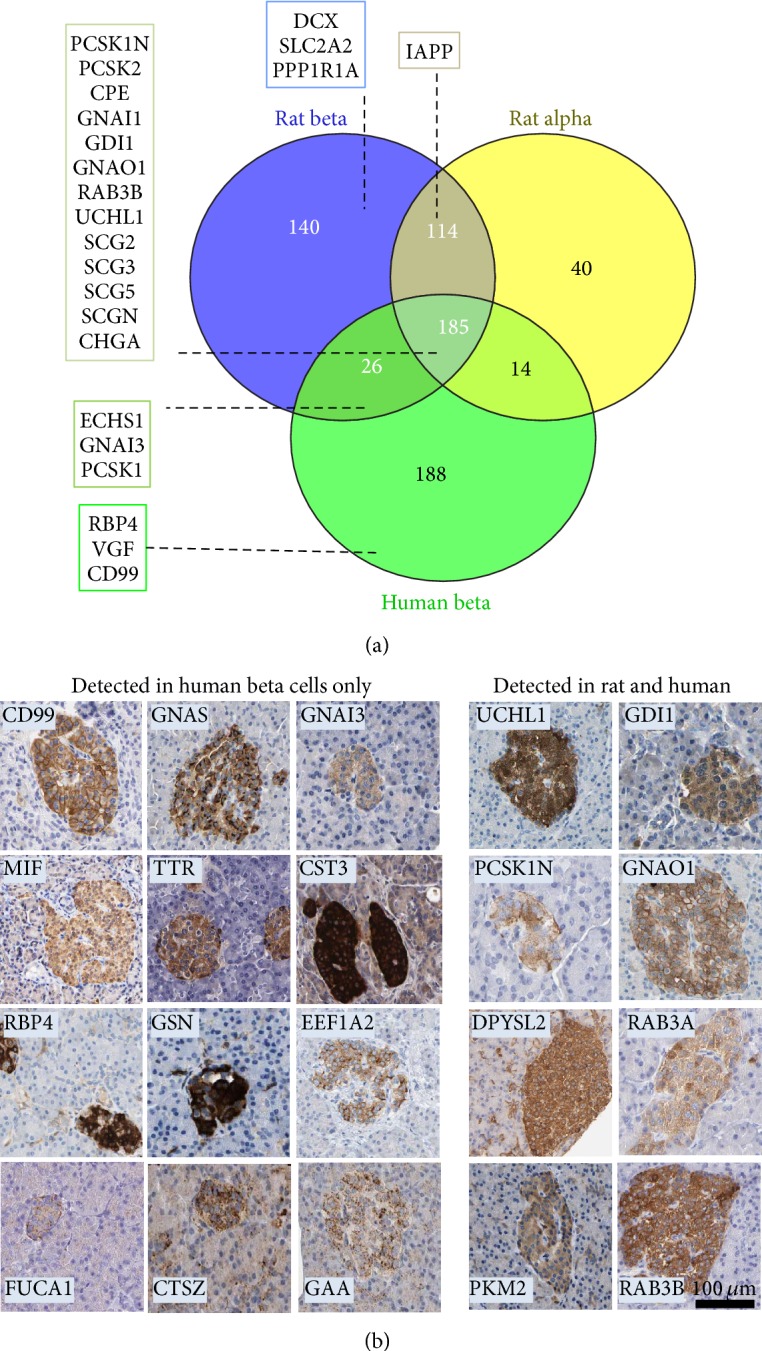
Overview of the LC-MS/MS data set and novel endocrine markers. (a) Venn diagram of number of proteins uniquely or commonly identified in human beta cells (*n* = 4) and rat alpha and beta cells (*n* = 3). Full list in Table S1. (b) Proteins with islet-restricted expression in pancreas according the Human Protein Atlas detected in both rat and human beta cells, or only in human beta cells.

Unfractionated protein extracts of freshly isolated rat beta (*n* = 3) and alpha (*n* = 3) cells, human islet endocrine cells FACS-enriched after culture (*n* = 4), and human exocrine cells (*n* = 1) were analyzed by alternate-scanning LC-MS/MS proteomics [15], resulting in the confident identification and relative quantification of 707 proteins (overview in Table 1 and Figure 1(a), full quantitative data included in Table S1). Apart from a majority of ubiquitous proteins, this data set confirmed the abundant expression of several established endocrine marker proteins in both rat and human beta cells (Figure 1(a), Table S1) but also revealed several other proteins that showed an islet-restricted expression in human pancreas as evidenced by the Human Protein Atlas [17]. Some of these novel candidate beta cell marker proteins were detected in both rat and human beta cells (Figure 1(b), right), but many others were confidently detected by LC-MS/MS in human cells but never in rat cells (Figure 1(b), left). The latter category includes plasma membrane protein CD99, secreted proteins MIF, TTR, and RBP4, tumor-marker EEF1A2, and several lysosomal enzymes (FUCA1, CTSZ, and GAA).

### 3.2. Proteome Architecture of Selected Core Pathways in Human and Rat Beta Cells

Relative molar abundances, as detected by label-free LC-MS/MS, were geometrically normalized using 6 reference proteins (MDH2, UBC, PPIA, HSPA8, PFN1, and CALM1), selected as described in Methods and Figure S1. This approach results in an accurate quantification of relative molar protein levels, directly comparable in both rat and human species, with <10% bias, as estimated by the techniques accurate measurement of multienzyme complex stoichiometry (Figure S2B).

### 3.3. Proteome Architecture of Intermediary Metabolism

**Figure 2:**
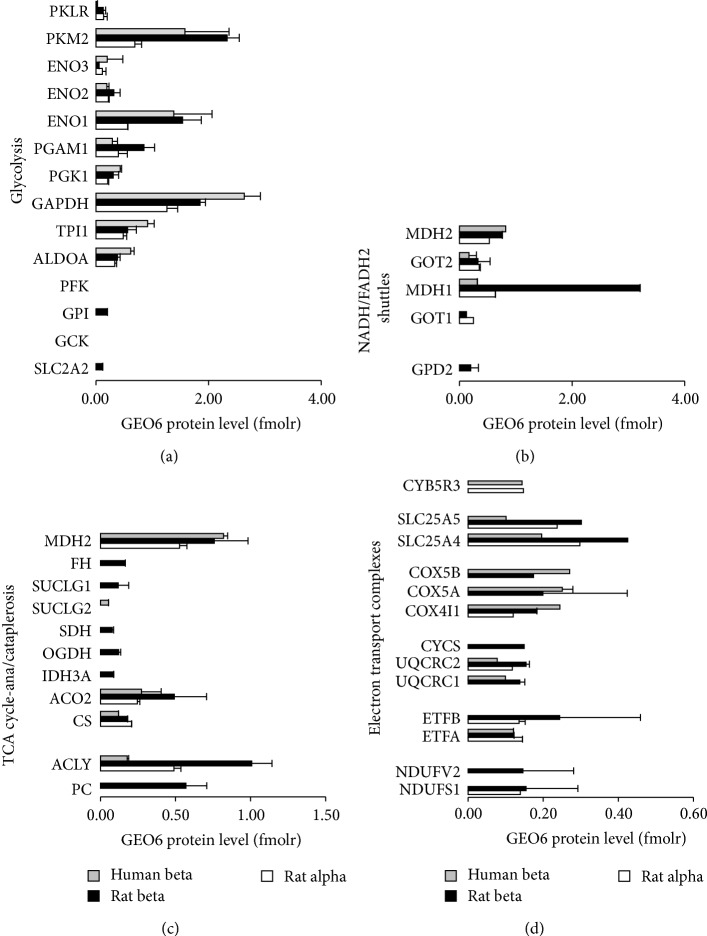
Proteome architecture of intermediary metabolic pathways in rat and human islet endocrine cells. Data represent mean ± SD geometrically normalized protein amounts in rat beta (black bars, *n* = 3), rat alpha (white bars, *n* = 3), and human beta cells (gray bars, *n* = 4). No error bar means confident detection in just 1 biological replicate. Proteins are denoted by their official gene symbol as recorded by UniProt (May 2014) and grouped for their main metabolic pathway: (a) glycolysis, (b) NADH/FADH2 shuttles. (c) Krebs cycle and ana- or cataplerosis. (d) Mitochondrial Electron Transport Complexes I–V. Full width at half maximum can be used as statistical discrimination limit for differential protein expression, corresponding to 2.35 times the median total error (19%) = 45%.

Human and rat beta cells showed an overall similar expression of glycolytic enzymes (Figure 2(a)), with higher levels of the enzymes of distal glycolysis. As compared to alpha cells, both rat and human beta cells expressed 2 times higher levels of enolase 1 (ENO1) and pyruvate kinase M2 (PKM2) isoform. Rat and human beta cells differed in their expression of cytoplasmic-to-mitochondrial carriers of reduced equivalents (Figure 2(b)): unlike human beta cells, rat beta cells showed very high levels of malate dehydrogenase 1 (MDH1), the cytosolic arm of the malate/aspartate NADH-shuttle, and had detectable expression of glycerol-phosphate dehydrogenase 2 (GPD2), the mitochondrial arm of the glycerol-phosphate FADH_2_-carrier. This is remarkable as these shuttles were previously found crucial for glucose-stimulated insulin secretion in rodents [18] but have never been studied in human beta cells. Krebs cycle enzymes (Figure 2(c)) such as aconitase 2 (ACO2), citrate synthase (CS), and reference protein MDH2 that could be quantified in both rat and human showed no or minor expression differences. A clear difference, however, was seen at the level of the cataplerotic enzyme ATP-citrate lyase (ACLY) and anaplerotic enzyme pyruvate carboxylase (PC) both more abundant in rat beta cells (Figure 2(c)); again this is remarkable as the anaplerotic flux of glucose-carbon through PC is considered an important regulator of glucose-stimulated insulin secretion based on rodent studies. At the level of the mitochondrial respiratory chain (Figure 2(d)), rat and beta cells expressed grossly similar amounts of complex IV (COX5A, COX5B, and COX4I1) and V (SLC25A5, SLC25A4; see also Figure 2(d)) subunits, but complex I (NDUFV2, NDUFS1), II (ETFB, ETFA), and III (CYCS, UQCRC2, UQCRC1) subunits were more abundant in rat beta cells. Human beta cells also express much more lysosome-associated proteins than rat cells (Table S1, Figure 1(b)) but much less enzymes involved in catecholamine production (Table S1).

### 3.4. Rat Beta Cells Express More MDH1 In Situ Than Human Beta Cells

**Figure 3:**
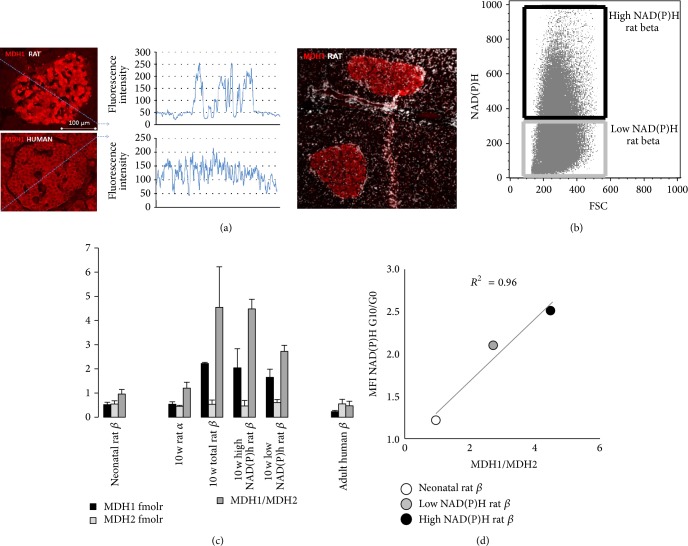
Beta cell-selective MDH1 expression in rat but not human pancreas. (a) A monoclonal antibody raised against recombinant human MDH1 fragment results in islet-restricted staining pattern in rat pancreas. In human pancreas, MDH1 fluorescence in islets does not exceed background level of surrounding exocrine tissue, as evidenced by intensity plotted along the dotted blue line, while in rat it shows a strong islet-restricted level. Representative of 3 organs analyzed for each species. (b–d) In rat, cellular MH1/MDH2 level increases during postnatal beta cell maturation from neonatal period to 10 weeks (c). In 10-week-old cells, beta cells FACS-sorted (b) for higher 7.5 mM glucose-induced NADH have higher MDH1/MDH2, and overall (d), the increment of cellular NADH acutely after 10 versus 0 mM glucose-stimulation is linearly correlated to cellular MDH1/MDH2 in the indicated rat beta cell phenotypes.

Analysis of MDH1 expression in pancreas, using a mouse monoclonal raised against a human recombinant MDH1 fragment, detected a clear MDH1 expression in beta cells/islets, well above the background level of surrounding exocrine pancreas (Figure 2(a)) in rat but not human pancreas, confirming the LC-MS/MS data. Of note, I previously observed that MDH1 expression in the rat beta cells is an indicator of postnatal beta cell maturation [12] and is more pronounced in rat beta cells with a higher intrinsic glucose-NADH metabolic responsiveness [10]. In particular, the ratio of MDH1/MDH2 expression appeared to be a reliable indicator of metabolic glucose responsiveness across different phenotypes in the rat beta cell (Figures 3(b)–3(d)).

### 3.5. Comparative Analysis of Heat Shock Proteins and Oxidative Stress Defense Pathways

**Figure 4:**
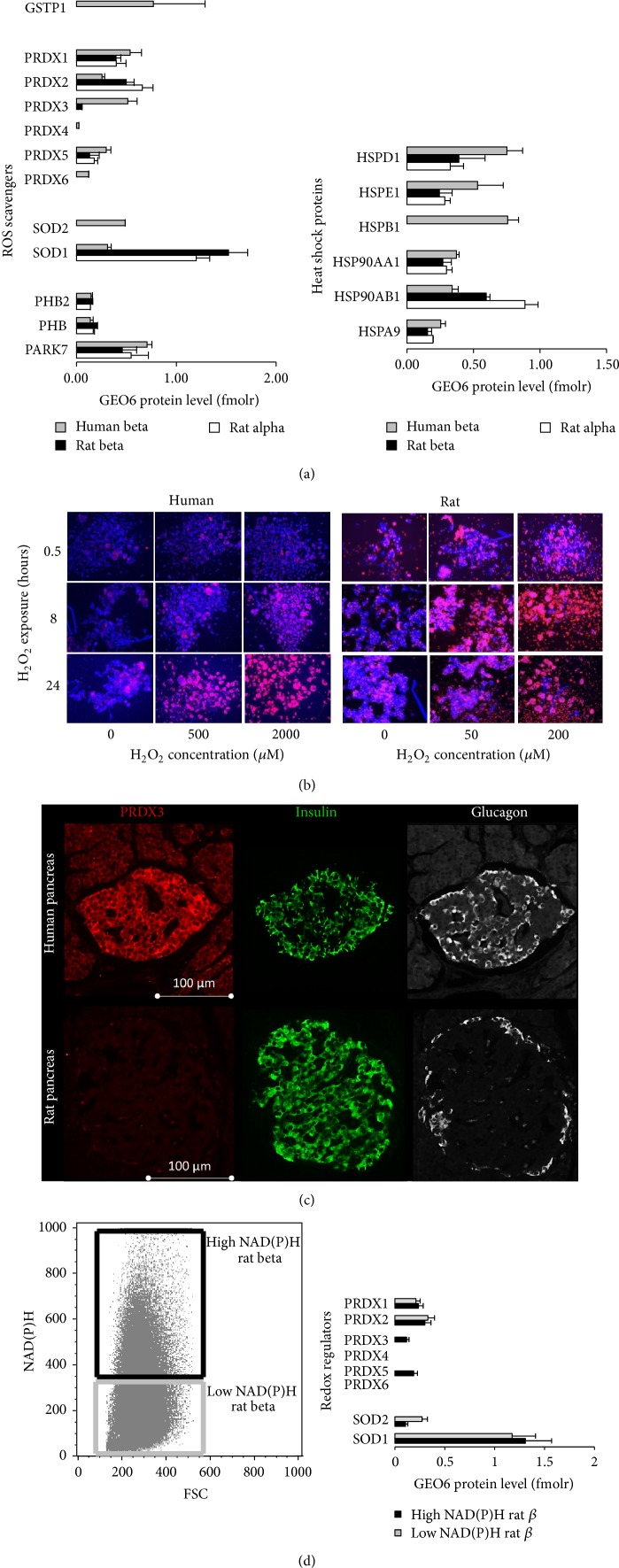
Differences between human and rat beta cells in oxidative stress/heat shock protein expression and response: higher expression of PRDX3 in human beta cells. (a) Overview graph of proteins associated to oxygen radical scavenging and stress-induced heat shock proteins. Data represent mean ± SD geometrically normalized protein amounts in rat beta (black bars, *n* = 3), rat alpha (white bars, *n* = 3), and human beta cells (gray bars, *n* = 4). (b) Human beta cells show a higher resistance to H_2_O_2_-induced cell death in vitro. Human and rat islets were exposed for 2 h to the indicated H_2_O_2_-concentrations and cell death was visualized by Hoechst-propidium iodide vital staining. (c) PRDX3 shows an islet-restricted pattern in human but not rat beta pancreas, as shown by immunofluorescence triple staining of human and rat pancreas for PRDX3 (red), insulin (green), and glucagon (white). (d) In rat beta cells, PRDX3 (and PRDX5) expression is restricted to beta cell subsets with higher glucose-NADH responsiveness as shown by quantitative LC-tandem MS. (Bars represent mean ± SD of *n* = 3 experiments, after geometric normalization for 6 reference proteins).

A comparison of stress-regulated/responsive enzymes in rat and human beta cells (Figure 4) confirmed the previously reported higher expression of heat shock protein 70 (HSPA9) expression in human as compared to rat beta cells [6, 8]. Human beta cells also expressed 2-times more heat shock 60 kD (HSPD1), 10 kD (HSPE1), and 27/28 kD (HSPB1) (Figure 4(a)). I additionally analyzed the defense pathways against reactive oxygen and nitrogen species (Figure 4(a)). Here too, I confirmed the previous observation [6, 8] of higher mitochondrial Mn superoxide dismutase expression (SOD2) in human versus rat beta cells: rat alpha and beta cells expressed 4 times more cytoplasmic SOD1 than human beta cells but showed no detectable SOD2. A novel observation was the clearly higher expression of peroxiredoxin family members in human beta cells. In particular, isolated human beta cells expressed 10 times higher levels of peroxiredoxin 3 (PRDX3), a highly potent mitochondrial scavenger of H_2_O_2_, peroxides, and peroxynitrite [19], also involved in physiological H_2_O_2_-signaling [20]. This fits with the known higher resistance of human beta cells to H_2_O_2_-induced cell death in vitro (Figure 4(b)).

### 3.6. Human Beta Cells Express More PRDX3 In Situ Than Rat Beta Cells

Using a polyclonal antibody raised against full-length human PRDX3, PRDX3 expression was compared in situ in rat and human pancreas (Figure 4(c)). In Western blotting, this antibody detected discrete bands corresponding to reduced sulfhydryl-PRDX3 (±18 kDa) and oxidized PRDX3 dimers (±36 kDa) in human tissues, with a weak (cross)reactivity to similar bands in rat tissues (not shown). PRDX3 staining of human pancreas revealed a clear islet-restricted staining pattern, which was not the case in rat pancreas where islets could not be discerned from background using this antibody. This does not imply that rat beta cells completely lack PRDX3: though 10-fold less abundant in the total rat beta cell population (Figure 4(a)), PRDX3 expression could confidently be detected by LC-MS/MS in rat cells, but more so in rat beta cells sorted for a higher metabolic glucose-NADH responsiveness (Figure 4(d) and [10]), in line with both the previously higher oxidative stress-resistance and lower endogenous peroxide production in these high responsive beta cell subsets [21].

## 4. Discussion

This study presents to our knowledge the first direct quantitative comparison of the core proteomic architecture of human and rat beta cells. Overall, it reveals a striking degree of conservation: for example, the relative molar abundance of most enzymes of glycolysis, Krebs cycle, beta-oxidation, and oxidative phosphorylation are quite similar. Attention is therefore drawn to a few exceptions, especially in their specialized intermediary metabolism and susceptibility to oxidative stress, suggesting biological differences not recognized thus far.

The main limitation of our study was the depth of proteome coverage: with the confident detection of 462 proteins, our coverage exceeds the 66 identifications previously reported using 2D-electrophoresis-MS in human islets [22] but lacks sensitivity as compared to the 3365 proteins identified by Metz et al. using 2D-LC-MS/MS [23]. The strength of our study resides in the use of label-free proteome quantification in combination with a careful normalization strategy, to allow a direct exploration of (dis)similarities in protein abundance between human and rat beta cells. As recently reviewed [24], both descriptive and quantitative proteomic techniques have been used to identify beta cell-selective markers, to characterize the beta cell's adaptation to diabetogenic stress conditions or physiological stimuli, and to characterize the beta cell's secretome [25]. Data independent alternate-scanning LC-MS/MS achieves reasonably accurate quantification, based on the observation that the average MS signal response for the three most intense tryptic peptides per mole of protein is constant within a coefficient of variation of less than +/−10% [14, 15]. In combination with our highly standardized protocols for preparing beta cells [26], this results in low total errors ≤20% [10]. Comparison of different biological replicates or different cell types requires the additional normalization of protein expression profiles to “housekeeping” or reference proteins. I applied a robust normalization strategy based on prior experience in normalization of mRNA expression levels quantified by real-time PCR [9, 10]. I selected a set of proteins that were detected in all human and rat biological replicates, at a stable relative level, indicating coregulation, and thus constitute a true “reference network.” This analysis revealed a conserved architecture of the “core proteome” of rat and human islet endocrine cells, consisting of the major metabolic routes, cell signaling scaffolds, and the cytoskeleton. Besides confirming the expected coregulation within functional pathways, I also observed a high degree of coregulation of molar abundance between different core pathways, adding biological meaning to our selection of a reference network. Though other combinations were possible, I ultimately handpicked a network consisting of usual suspects (PPIA, UBC) as well as more novel (CALM1, PFN1, MDH2, and HSPA8) reference proteins, selected from different functional pathways to provide a holistic representation of the cell. Geometric normalization [9] to this network resulted in the accurate (bias <10%) measurement, as illustrated by the identical subunit stoichiometry of the mitochondrial F0F1 ATPase both in rat and human beta cells, suggesting that quantitative dissimilarities are meaningful. Evidently, when directly comparing molar abundances in rat and human beta cell preparations, caution is warranted in view of the ±5 times higher number of contaminating glucagon-expressing cells in the human than rat beta cell preparations, and allocation of observed differences can only be done in combination with in situ studies of beta cell-selective protein expression.

Dissimilarities were found in key glucose-sensing pathways, shown to be important for glucose-stimulated insulin secretion (GSIS) in rodents. Human beta cells express 10-fold less cytoplasmic MDH1 but equal amounts of mitochondrial MDH2 than rat beta cells. They also express less electron transport chain complex I subunits, the main site of NADH regeneration. This is remarkable because MDH1 is required for the malate/aspartate shuttle considered crucial for GSIS in rodents [27], and because in rodents a low MDH1/MDH2 ratio was proposed as sign of incomplete beta cell maturation [12, 28]. Another noticeable finding is the higher expression of pyruvate carboxylase (PC) by rat beta cells. By converting pyruvate to oxaloacetate, PC feeds new carbon carriers into the Krebs cycle, ready to accept acetyl-CoA destined towards aerobic oxidation. In rodents, this anaplerotic flux was quantitatively important and considered crucial for GSIS [29].

Long-lived, postmitotic cells, such as the human beta cell, need a strong antioxidative defense or vice versa; the gradual overproduction of oxygen radicals in aging cells induces upregulation of antioxidant defenses in surviving cells [30]. It is since long time known that isolated human beta cells are far more resistant to nitrergic and oxidative stresses than rodent beta cells [7]; this difference was previously attributed to a higher expression of heat shock proteins, catalase, and SOD [8]. Here I confirm this but also identify PRDX3 (and PRDX5) as possible new regulators. It is unlikely that these upregulations are an artifact induced by stressful beta cell isolation because, though PRDX3 is ubiquitously expressed, in situ analysis indicated a higher PRDX3 immunoreactivity in human islets than in the surrounding exocrine pancreas tissue. Though clearly less abundant, PRDX3 was also detectable by LC-LM/MS in rat beta cells, but not uniformly. Rat beta cells show intercellular differences in their metabolic glucose sensitivity. Beta cell subsets characterized by a higher expression of glucokinase and its downstream glycolytic enzymes [10, 31] produce more NAD(P)H and less oxygen radicals after stimulation by glucose [21]. Highly responsive beta cell subsets also secrete more insulin and show a higher resistance to oxidative stress-induced apoptotic cell death. Here I show that these cells also express higher levels of peroxiredoxins PRDX3 and PRDX5, but not SOD2. Physiological variations in the rat beta cell's intrinsic glucose-regulated redox state are thus correlated to variations in their PRDX3 expression. Future studies will have to address the specific role of enzymes such as PRDX3 in loss- or gain-of function in vitro readouts. Yet, since PRDX3 is a highly efficient intramitochondrial H_2_O_2_ scavenger [19, 20] that operates optimally at physiological intramitochondrial H_2_O_2_ concentrations [20], it is clearly an attractive readout for future functional studies on glucose-regulated redox signaling.

In conclusion, current study provides a first quantitative proteome comparison of unfractionated rat and human islet endocrine cells. Though the quantitative architecture of their core proteome is highly conserved, rat and human beta cells show remarkable differences in their specialized nutrient-sensing pathways, lysosomal compartments, and oxidative stress that are compatible with the proteomic adaptation of the human beta cell as a long-lived, oxidative stress-resistant cell type.

## Supplementary Material

Supplementary Table S1: Overview of all proteins identified and/or quantified in rat and human pancreatic cell types.
Supplementary Figure S1: Selection of a reference protein network for geometric normalization.
Supplementary Figure S2: Accuracy of molar quantification of geometrically normalized LC-MS/MS data.

## Figures and Tables

**Table 1:** Overview of proteome coverage by LC-MS/MS.

	Rat		Human	
	Beta	Alpha	Endocrine	Exocrine
Number of proteins identified				
n ≥ 1	506	417	462	372
Number of proteins quantified				
n ≥ 2	465	353	413	300
GEO-normalized molar amount				
Highest	3.61	8.22	8.95	14.74
Lowest	0.02	0.02	0.01	0.06
Dynamic range	226	548	746	246
Biological replicates (x)	3	3	4	1
Technical replicates (n)	3	3	3	3

Unfractionated protein extracts of FACS-purified pancreatic beta and alpha cells (*n* = 3) and FACS-enriched human islet endocrine cells (*n* = 4) were analyzed by alternate-scanning LC-MS/MS. As additional quality control, one pancreatic exocrine cell-enriched human sample was also measured. All samples were injected in triplicate, yielding 372 (300) to 506 (465) proteins that met the criteria for identification (quantification, if detected in *n* ≥ 2 of 3 technical replicates) as specified in Methods, with dynamic ranges (difference between the lowest and the highest quantifiable concentration) ranging from 226 to 746 as listed.

## Abbreviations

FACS: Fluorescence-activated cell sorting
GSIS: Glucose-stimulated insulin secretion
LC-MS/MS: Liquid chromatography-tandem mass spectrometry.
