# Supplementary figures and images for: Species-Related Differences in the Proteome of Rat and Human Pancreatic Beta Cells
Source: J Diabetes Res. 2015 May 10;2015:549818. doi: 10.1155/2015/549818 (PMC4442007; doi:10.1155/2015/549818)

1  
2 **Fig. S1**

A

Proteins quantified in all biological replicates

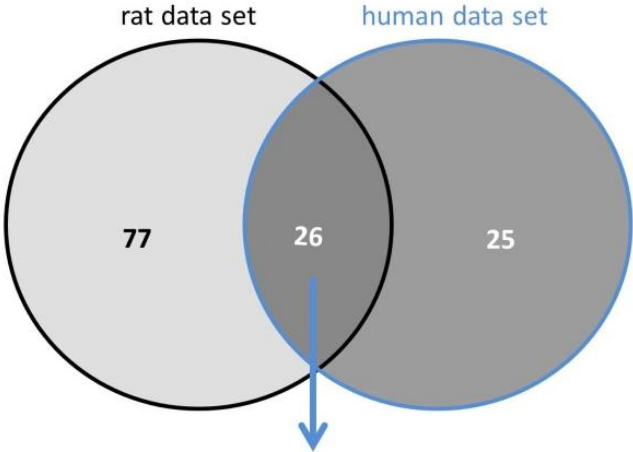

B

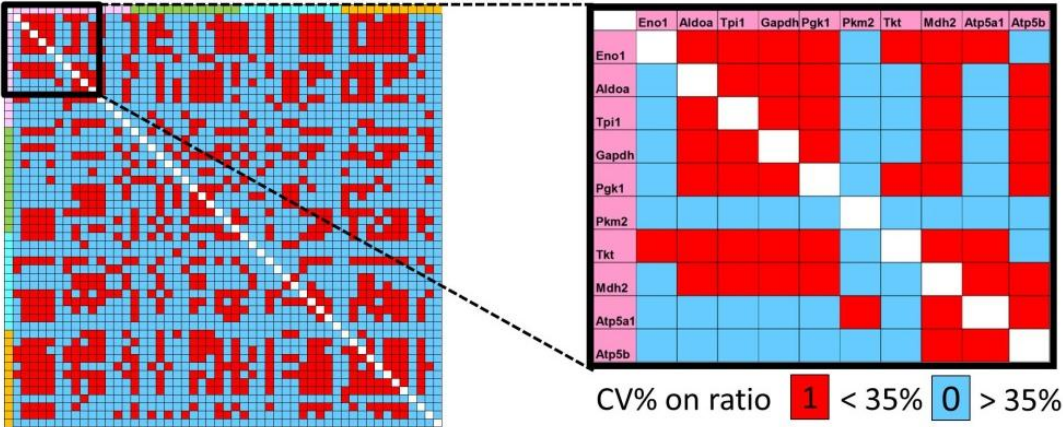

3  
4

1 Fig. S1

C

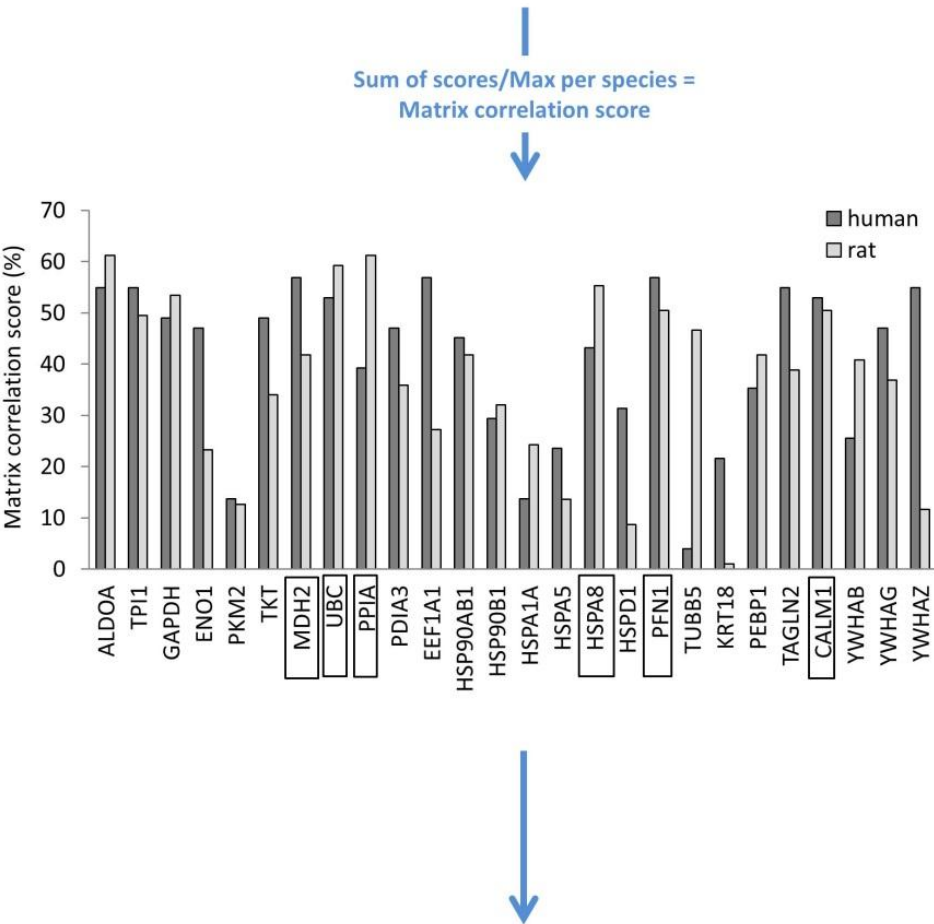

2

3

1 Fig. S1

D

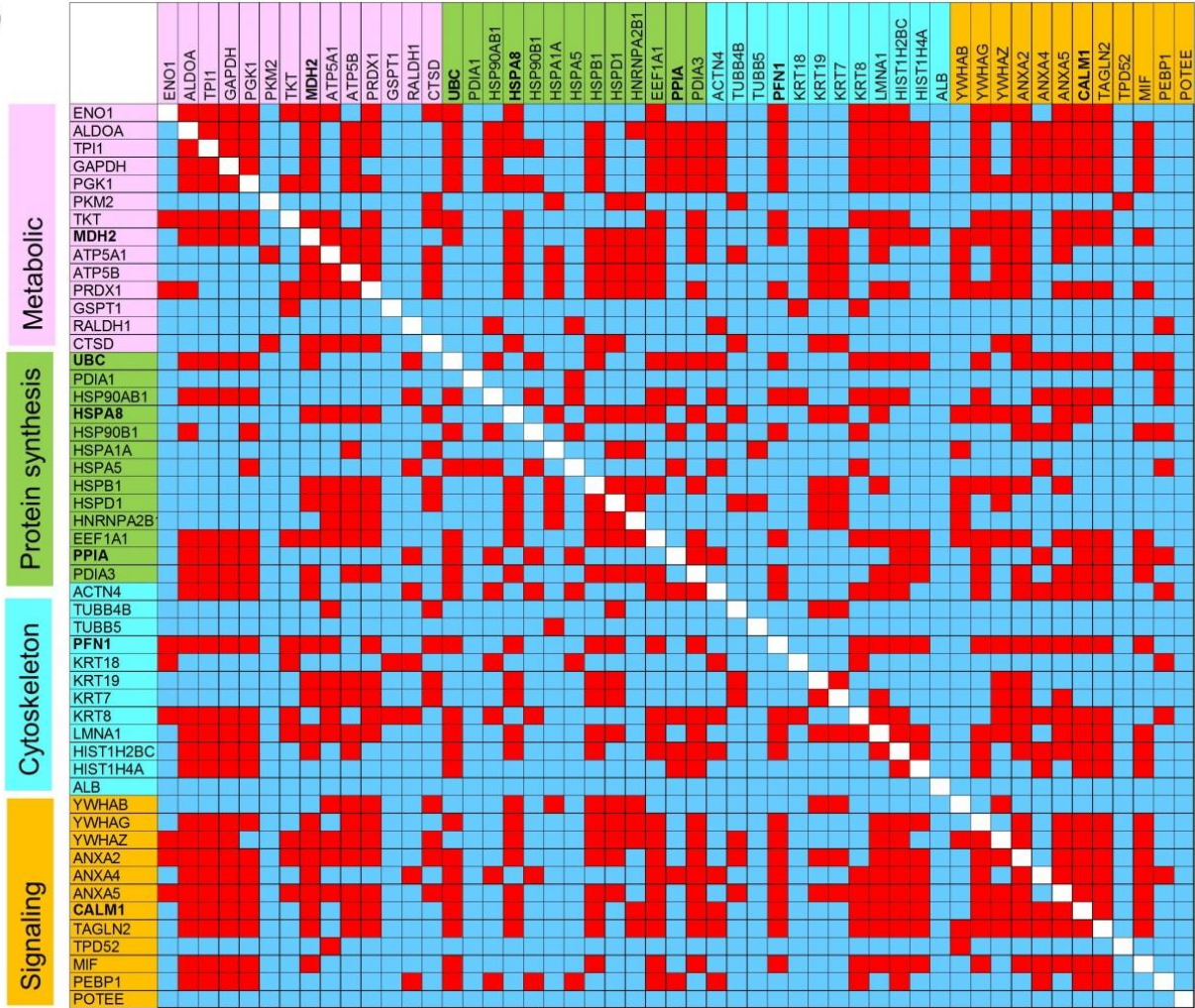

2

3

1 Fig. S2

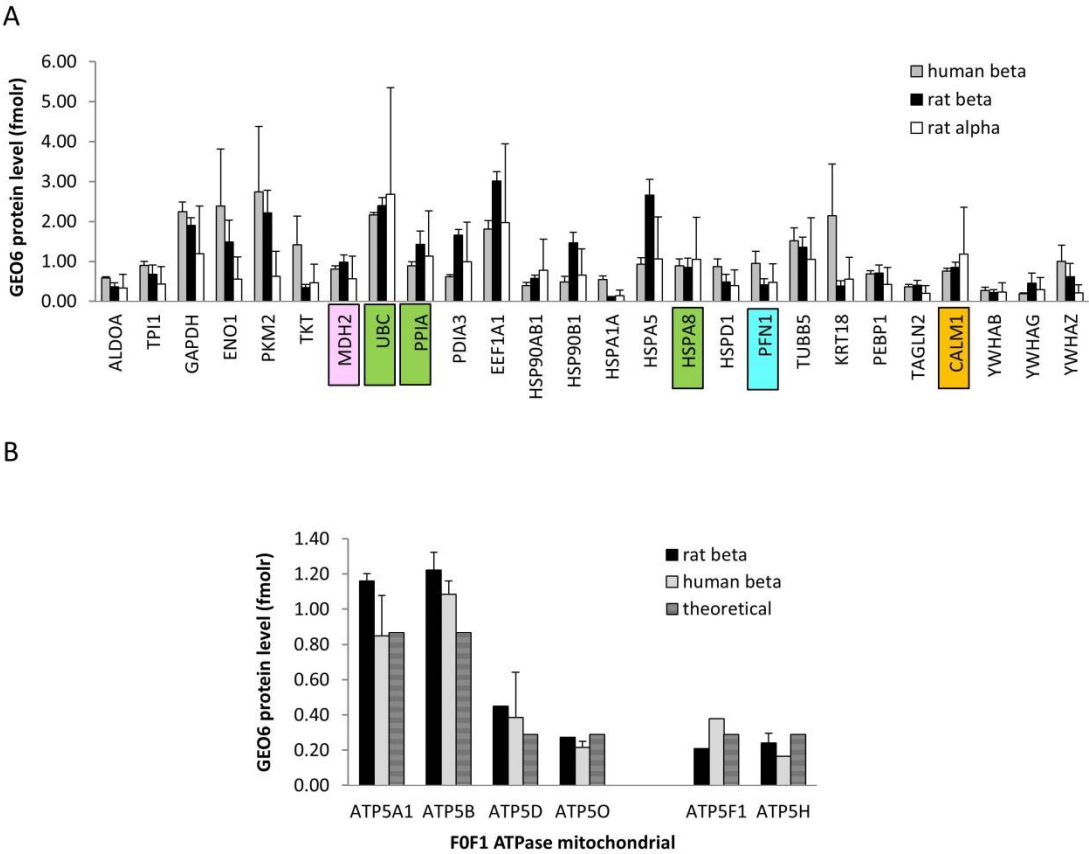

2

Supplement: Supplementary file 1 — Supplementary Table S1: Overview of all proteins identified and/or quantified in rat and human pancreatic cell types. Supplementary Figure S1: Selection of a reference protein network for geometric normalization. Supplementary Figure S2: Accuracy of molar quantification of geometrically normalized LC-MS/MS data. [file 549818.f1.zip › Supp. Figures.pdf]
